# Efficient terminal erythroid differentiation requires the APC/C cofactor Cdh1 to limit replicative stress in erythroblasts

**DOI:** 10.1038/s41598-022-14331-6

**Published:** 2022-06-21

**Authors:** Myriam Cuadrado, Javier Garzón, Sergio Moreno, Irene García-Higuera

**Affiliations:** 1 Instituto de Biología Funcional y Genómica (IBFG), CSIC/Universidad de Salamanca, C/Zacarías González 2, 37007 Salamanca, Spain; 2 Instituto de Biología Molecular y Celular del Cáncer, CSIC/Universidad de Salamanca, 37007 Salamanca, Spain; 3 Departamento de Biología Molecular, Instituto de Biología Molecular (IUBM) and Centro de Biología Molecular “Severo Ochoa”, Universidad Autónoma de Madrid/CSIC, C/Nicolás Cabrera 1, 28049 Madrid, Spain; 4 Present Address: Adrestia Therapeutics, Babraham, Cambridge, CB22 3AT UK

**Keywords:** Ubiquitin ligases, Cell-cycle proteins, Cell division, Genomic instability, Differentiation

## Abstract

The APC/C-Cdh1 ubiquitin ligase complex drives proteosomal degradation of cell cycle regulators and other cellular proteins during the G1 phase of the cycle. The complex serves as an important modulator of the G1/S transition and prevents premature entry into S phase, genomic instability, and tumor development. Additionally, mounting evidence supports a role for this complex in cell differentiation, but its relevance in erythropoiesis has not been addressed so far. Here we show, using mouse models of Cdh1 deletion, that APC/C-Cdh1 activity is required for efficient terminal erythroid differentiation during fetal development as well as postnatally. Consistently, Cdh1 ablation leads to mild but persistent anemia from birth to adulthood. Interestingly, loss of Cdh1 seems to affect both, steady-state and stress erythropoiesis. Detailed analysis of Cdh1-deficient erythroid populations revealed accumulation of DNA damage in maturing erythroblasts and signs of delayed G2/M transition. Moreover, through direct assessment of replication dynamics in fetal liver cells, we uncovered slow fork movement and increased origin usage in the absence of Cdh1, strongly suggesting replicative stress to be the underlying cause of DNA lesions and cell cycle delays in erythroblasts devoid of Cdh1. In turn, these alterations would restrain full maturation of erythroblasts into reticulocytes and reduce the output of functional erythrocytes, leading to anemia. Our results further highlight the relevance of APC/C-Cdh1 activity for terminal differentiation and underscore the need for precise control of replication dynamics for efficient supply of red blood cells.

## Introduction

Erythropoiesis is a complex differentiation process that drives production of red blood cells in the fetal liver of developing embryos or in the bone marrow of adult individuals^1^. Hematopoietic stem cells give rise to committed erythroid progenitors that progressively differentiate and produce proerythroblasts, thus initiating terminal erythroid differentiation. Proerythroblasts undergo 3 to 4 mitotic cell divisions while sequentially maturing into basophilic, polychromatic, and orthochromatic erythroblasts that, in turn, become reticulocytes following cell cycle exit and enucleation. Additional morphological changes and organelle clearance yield fully functional erythrocytes^2,3^. All these events must be tightly regulated to ensure an adequate supply of red blood cells for tissue oxygenation under normal and stress conditions. Moreover, a strict coordination between proliferation and differentiation during terminal erythroid maturation is critical to prevent anemia, a condition associated with poor clinical outcomes and decreased quality of life^4,5^. In this regard, identifying new players involved in this process and further addressing their mechanistic contribution could set the grounds for new therapeutic strategies to treat genetic or acquired red blood cell disorders.

The Anaphase-Promoting Complex/Cyclosome (APC/C) is a multi-subunit E3-ubiquitin ligase that controls the cell division cycle by targeting specific substrates for proteosomal degradation, thus ensuring unidirectional and irreversible cell cycle progression^6,7^. In addition to the 14 different proteins present in the core complex, two alternative cofactors are required to keep APC/C active during mitosis and the G1 phase: Cdc20 and Cdh1 (also known as Fzr1 in mammals). The APC/C-Cdc20 complex forms at the onset of mitosis and triggers sister chromatid separation by targeting securin for proteosomal degradation, thus facilitating the release of separase to promote cohesin proteolysis. At the end of the mitotic phase Cdh1 takes over and sustains APC/C activity throughout the following G1 phase, driving proteolysis, among others, of mitotic cyclins and kinases, replication factors, and the F-box protein Skp2 that, in turn, promotes SCF-mediated ubiquitination of the Cdk inhibitors p27, p21 and p57^8,9^. Therefore, while Cdc20 is essential for cell division, APC/C-Cdh1 is a negative cell cycle regulator that restrains Cdk activity and preserves the G1 phase to allow proper preparation for a new round of DNA replication^10,11^. Accordingly, Cdh1 is not required for cell proliferation, but human and mouse cells devoid of Cdh1 show a shortened G1 phase and premature entry into S phase^12–16^. Moreover, we recently demonstrated in Cdh1-deficient MEFs slow replication fork progression and replicative stress that result in accumulation of DNA breaks and genomic instability^17^. APC/C-Cdh1 also seems to be important for cell cycle exit, particularly during differentiation^18^. Loss of Cdh1 in *Drosophila* embryos prevents epidermal cells from arresting after the terminal mitosis and induces supplementary cellular divisions^19^. Likewise, we and others have previously reported delayed exit of mouse neural progenitors from the cell cycle in the absence of Cdh1, leading to replicative stress, apoptotic cell death and impaired neurogenesis in vivo^20,21^. Finally, spermatogenesis was also found to be affected in conditional mouse models of Cdh1 deletion^22^ and defective myoblast and lens differentiation were described in in vitro cell systems upon Cdh1 knockdown^23,24^.

Given the various precedents implicating APC/C-Cdh1 in several differentiation events, in the current study we set out to investigate if erythropoiesis, the well-defined process of erythroid maturation, could also rely on this ubiquitin ligase complex. Using Cdh1-deficient mouse embryos and adult mice specifically depleted of Cdh1 in the hematopoietic lineage, we uncovered inefficient terminal erythroid differentiation during fetal and adult erythropoiesis in the absence of Cdh1 resulting in persistent mild anemia from birth to adulthood. Importantly, these phenotypes are accompanied by signs of replicative stress and accumulation of DNA damage in maturing erythroblasts, suggesting that aberrant replication and the resulting DNA breakage underlie defective erythroid development. These findings further highlight the relevance of tight control of cell cycle progression for optimal production of red blood cells and underscore the role of timed proteolysis for such regulation.

## Results

### Defective erythropoiesis in Cdh1-deficient embryos

**Figure 1:**
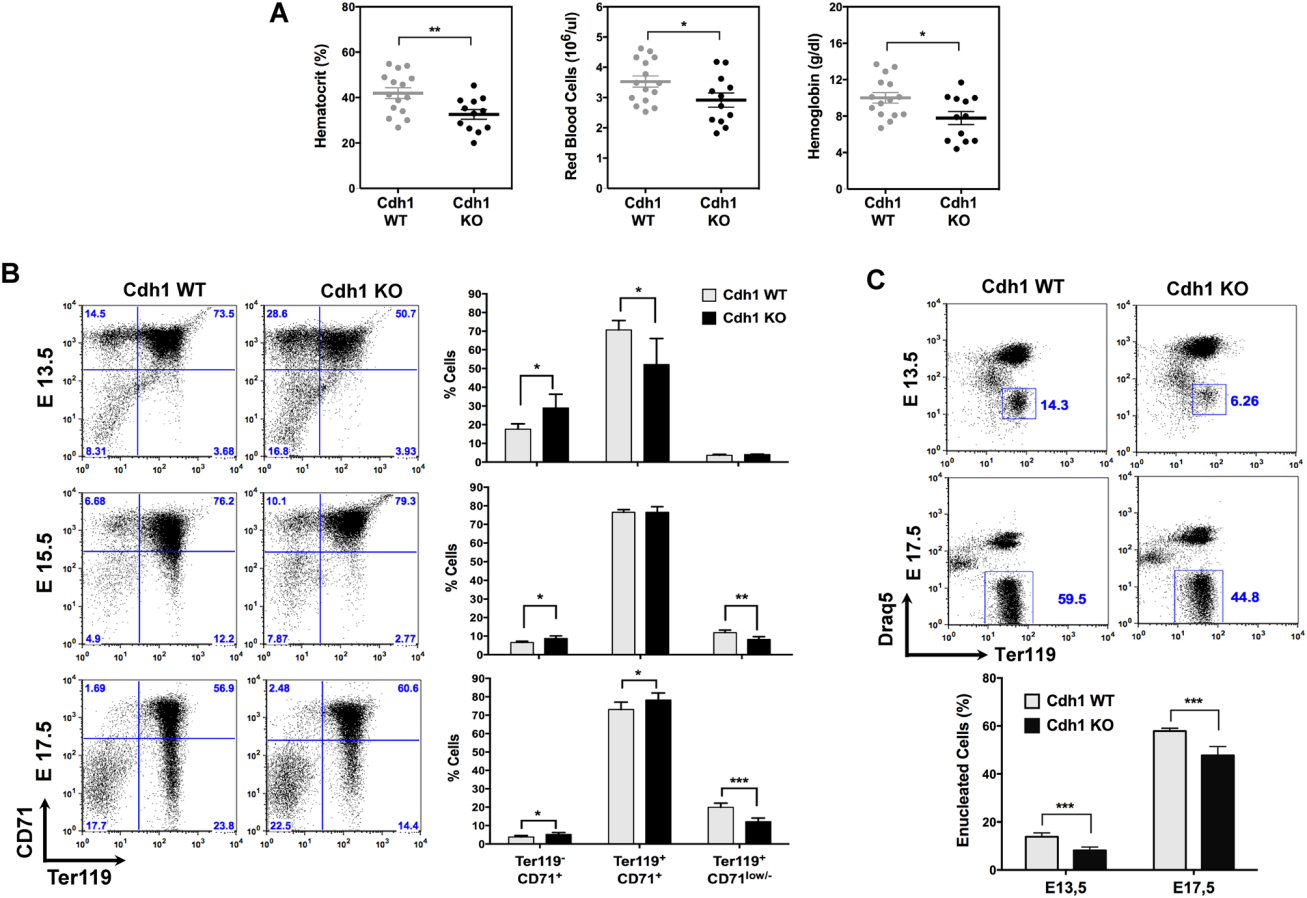
Cdh1 deficiency leads to inefficient fetal erythropoiesis and anemia at birth. (**A**) Red cell parameters from peripheral blood of control (Cdh1 WT) and Cdh1-deficient (Cdh1 KO) pups collected 12 to 18 h after birth. The scatter dot plots show mean ± s.e.m of the corresponding data (n = 15 for WT and 12 for KO). (**B**) Erythroid maturation was assessed based on expression of Ter119 and CD71 cell surface markers in fetal livers of the indicated embryonic stage and genotype. The ratios of pro-erythroblasts (Ter119^-^ CD71^+^), immature erythroblasts (Ter119^+^ CD71^+^), and mature erythroblasts and reticulocytes (Ter119^+^ CD71^low/-^) were determined in 4–7 samples for each stage and genotype (bar graphs, right). Representative flow cytometry plots are included on the left. (**C**) Quantification of the percentage of enucleated erythroid cells (Ter119^+^ Draq5^-^) in fetal livers from the indicated embryonic stage and genotype. The top panels show representative flow cytometry profiles. n = 10 for E13.5; n = 6 for E17.5; *P < 0.05, **P < 0.01, ***P < 0.001.

We have previously shown that expression of Cdh1 in embryonic tissues is not essential for mouse development, but Cdh1-null pups die 24 to 48 h after birth. Therefore, to assess whether or not the APC/C-Cdh1 ubiquitin ligase complex is important for red cell production, we first performed hematological analyses on blood samples from Cdh1-proficient (Cdh1 WT) and Cdh1-deficient (Cdh1 KO) newborn mice. Reduced hematocrit values, red blood cell counts, and hemoglobin concentration were found in Cdh1 KO pups (Fig. 1A), indicating mild anemia in the absence of Cdh1 and suggesting a role for APC/C-Cdh1 in erythropoiesis. To further explore this possibility we turned to the liver of developing embryos, as it is the predominant erythropoietic organ from mid-gestation onwards and even during the first days of postnatal life. We compared erythroid differentiation in wild type and Cdh1-null fetal liver by analyzing expression of the cell surface markers Ter119 and CD71 as previously described^25^. As shown in Fig. 1B, the ratio of Ter119^−^ CD71^+^ proerythroblasts was increased in Cdh1-deficient fetal livers at E13.5, while that of Ter119^+^ CD71^+ ^immature erythroblasts was decreased. A reduction in the percentage of Ter119^+^ CD71^low/-^mature erythroblasts and reticulocytes was also evident at later gestational stages (E15.5, E17.5), consistent with delayed or inefficient terminal erythroid differentiation in the absence of Cdh1. These observations were confirmed when we quantified the percentage of enucleated erythroid cells (Draq5^−^ Ter119^+^) and found significantly diminished values in Cdh1 KO fetal livers at E13.5 and E17.5 (Fig. 1C). These results point to the APC/C-Cdh1 ubiquitin ligase complex as a relevant player for efficient fetal erythropoiesis.

### Specific Cdh1 deletion in hematopoietic cells leads to inefficient fetal and adult erythropoiesis and anemia

**Figure 2:**
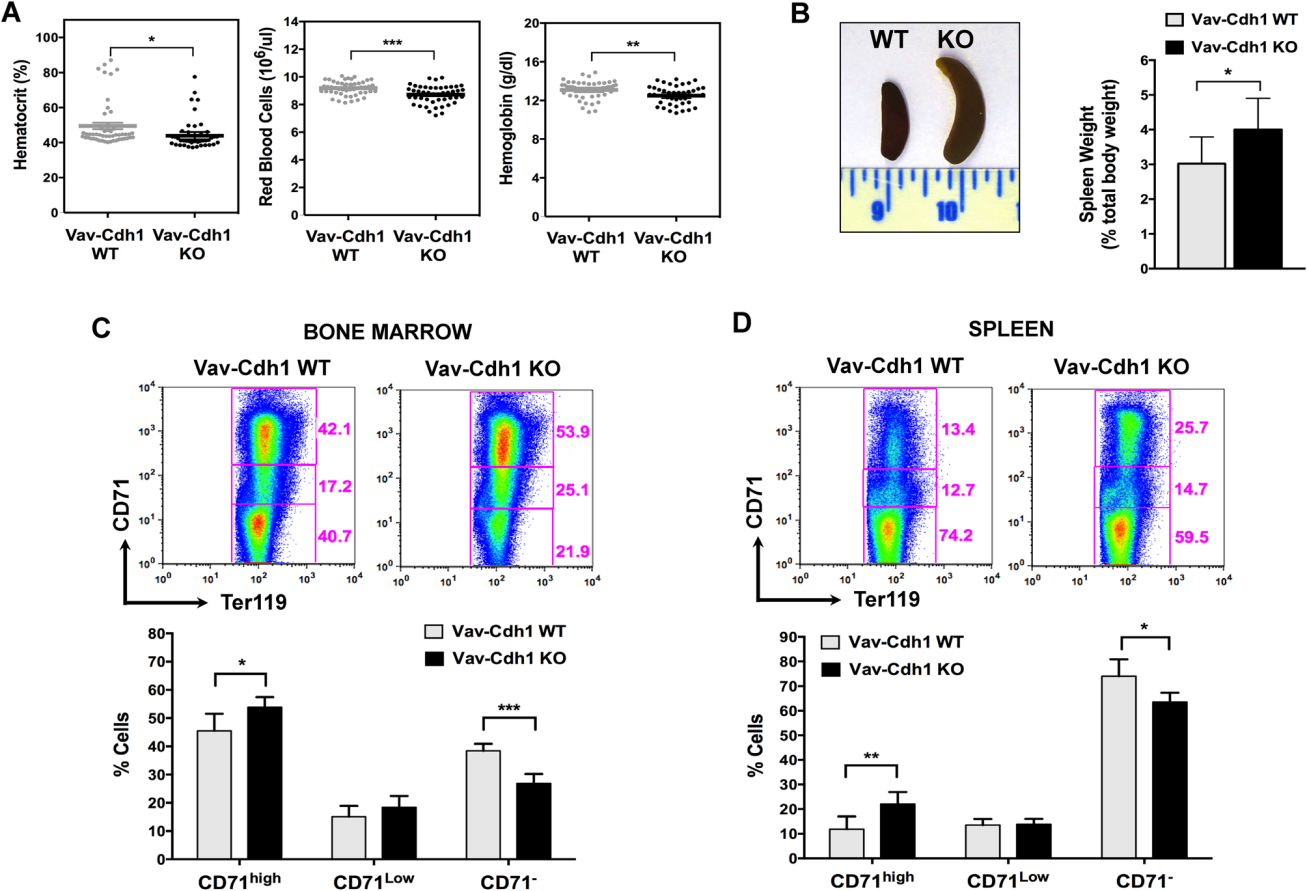
Signs of anemia and impaired terminal erythroid differentiation in adult mice lacking Cdh1 expression in hematopoietic cells. (**A**) Red cell parameters from peripheral blood of 8–12 week old mice of the indicated genotype. The scatter dot plots show mean ± s.e.m of the corresponding data. (n = 51 for Vav-Cdh1 WT and 46 for Vav-Cdh1 KO). (**B**) Relative weight of the spleen in control (Vav-Cdh1 WT) and mutant (Vav-Cdh1 KO) adult mice (n = 10). A representative picture is included (left). (**C**, **D**) Terminal erythroid maturation was assessed based on CD71 expression in Ter119^+^ gated cells in the bone marrow (**C**) and spleen (**D**) from adult mice of the indicated genotype. The relative abundance of populations showing high, low, or no CD71 expression, representing increasingly higher maturation stages, were quantified (bottom graphs). Representative flow cytometry plots are included (top panels). (n = 6). *P < 0.05, **P < 0.01, *** *P* < 0.001.

To rule out a general developmental delay as the cause of defective erythroid maturation in Cdh1-null embryos, we specifically deleted Cdh1 in hematopoietic stem cells (HSCs) using a *Vav1-Cre* transgene^26^. Significant downregulation of Cdh1 mRNA (Fig. S1A) and protein (Fig. S1B) levels was confirmed in blood cells and hematopoietic tissues from *Cdh1*^*fl/fl*^* Vav1-Cre* mice (Vav-Cdh1 KO) compared with *Cdh1*^*fl/fl*^ animals (Vav-Cdh1 WT). We first tested whether or not newborn mice lacking Cdh1 expression only in hematopoietic cells could recapitulate the mild anemia phenotype observed in Cdh1-null pups. Indeed, lower values for hematocrit, red blood cell number, and hemoglobin concentration were found in blood samples from Vav-Cdh1 KO newborns (Fig. S1C). Likewise, erythropoietic differentiation was also similarly affected in fetal liver from Vav-Cdh1 KO embryos, that showed accumulation of immature erythroblast populations (Ter119^+^ CD71^+^) and shortage of mature erythroblasts and reticulocytes (Ter119^+^ CD71^low/−^) (Fig. S1D). These observations support a specific role for Cdh1 during fetal erythropoiesis. Analysis of blood parameters in young adult mice further substantiated reduced red cell numbers upon hematopoietic Cdh1 deletion (Fig. 2A). Moreover, Vav-Cdh1 KO animals showed enlarged spleen (Fig. 2B), commonly associated with compensatory extramedullary erythropoiesis, and consistent with persistent anemia. To verify if, as suggested by this initial phenotypic characterization, Cdh1 is also relevant for adult erythropoiesis, we assessed expression of the transferrin receptor (CD71) in Ter119-positive cells in bone marrow (Fig. 2C) and spleen (Fig. 2D) of control and mutant mice. Both hematopoietic tissues depleted of Cdh1 displayed a moderate enrichment in immature erythroblastic populations (Ter119^+^ CD71^high^) accompanied by a concomitant decline in the most mature erythroid populations (Ter119^+^ CD71^−^), confirming inefficient maturation of erythroblasts in the absence of Cdh1 also in adulthood.

### Cdh1 is also required for efficient stress erythropoiesis

**Figure 3:**
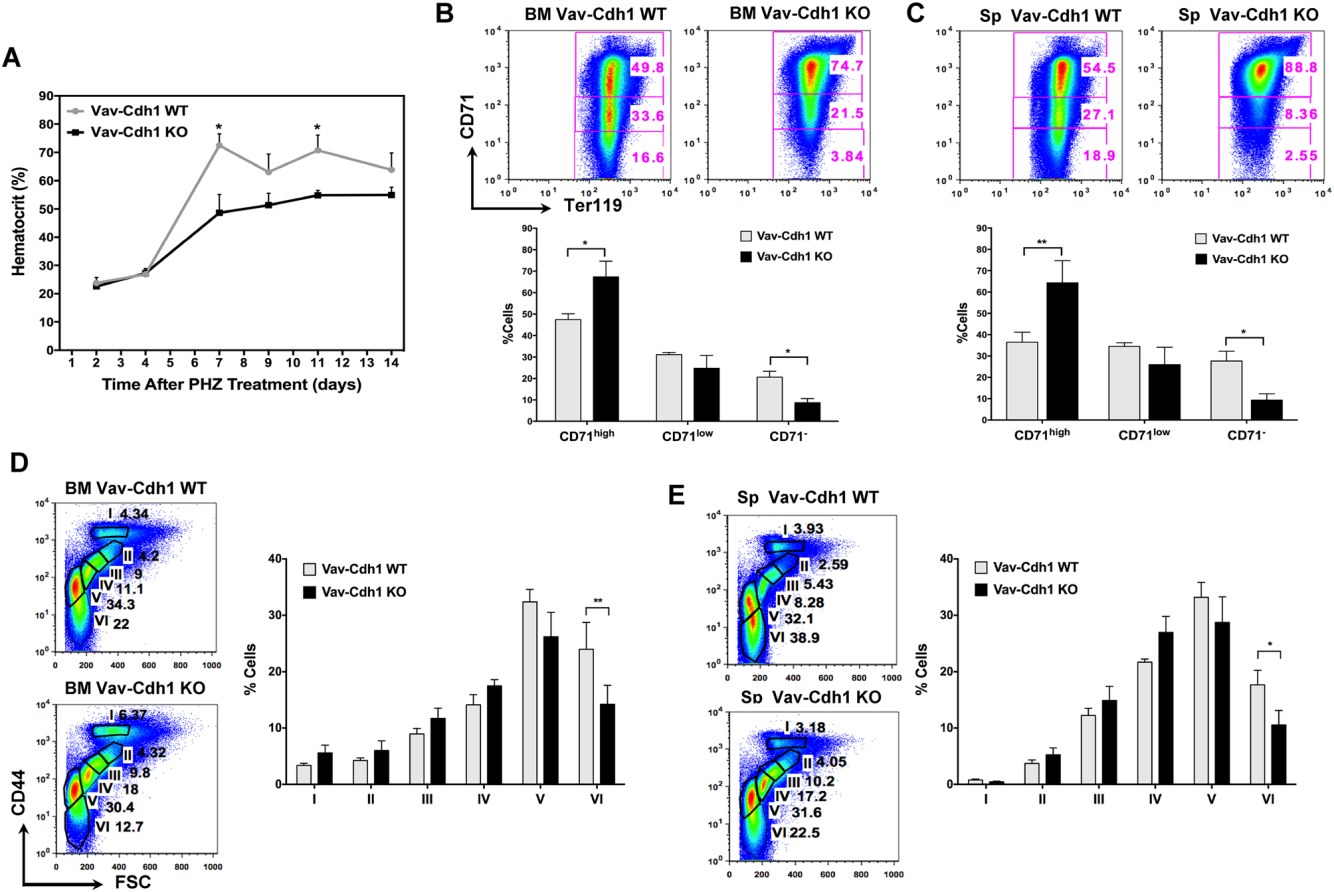
Slow recovery from hemolytic anemia and inefficient stress erythropoiesis in hematopoietic Cdh1-depleted mice. (**A**) Hematocrit values in control (Vav-Cdh1 WT, grey) and Cdh1-depleted (Vav-Cdh1 KO, black) animals at day 2, 4, 7, 9, 11 and 14 following PHZ injection. Each point represents the mean ± s.e.m of values obtained from 5 different animals of the corresponding genotype. (**B**, **C**) Seven days after PHZ treatment mice were sacrificed and terminal erythroid maturation was assessed in bone marrow (**B**) and spleen (**C**) based on CD71 expression in Ter119^+^ gated cells. The relative abundance of populations showing high, low, or no CD71 expression, representing increasingly higher maturation stages, was quantified (bottom graphs). Representative flow cytometry plots are included (top panels). (**D**, **E**) CD44-FSC (Forward Scatter) based staging of erythroid populations in the bone marrow (**D**) and spleen (**E**) of PHZ-treated mice of the indicated genotype after seven days of recovery. Population I corresponds to proerythroblasts, II, III and IV to basophilic, chromatophilic and orthochromatic erythroblasts, and V and IV to enucleated reticulocytes and erythrocytes. Representative flow cytometry profiles are shown in the top panels. The numbers included in the plots indicate the percentage of each population within erythroid cells (Ter119 positive). *P < 0,05, **P < 0.01, ***P < 0.001.

We next sought to determine if, in addition to its role in steady-state erythropoiesis, Cdh1 was also important during stress erythropoiesis. To that end we exposed Vav-Cdh1 WT and Vav-Cdh1 KO mice to Phenylhydrazine (PHZ) to induce hemolytic anemia^27–29^, and monitored their recovery during two weeks. As summarized in Fig. 3A, normal hematocrit values were restored at day 7 following PHZ injection in control mice, while Cdh1-depleted animals recovered slower and showed lower hematocrit values at all times. Moreover, terminal erythroid differentiation was also impaired in bone marrow (Fig. 3B) and spleen (Fig. 3C) from mutant animals recovering from hemolytic injury, where, once again, we detected an increased ratio of immature erythroblasts (Ter119^+^ CD71^high^) and a parallel reduction in the ratio of mature erythroid cells (Ter119^+^ CD71^−^). A more detailed analysis based on expression of the cell surface marker CD44 and cell size, that allows separation of six erythroid subpopulations (I to VI)^30^, confirmed both in bone marrow (Fig. 3D) and spleen (Fig. 3E), a decreased presence of the later stage populations, erythrocytes (VI) and reticulocytes (V) and a tendency to accumulate earlier stage precursors, namely basophilic (II), polychromatic (III) and orthochromatic (IV) erythroblasts. These observations support a wider, more general role for Cdh1, that would be required to ensure efficient production of erythrocytes to maintain red blood cell homeostasis under basal physiological conditions, but also to facilitate recovery from stress situations such as hemolytic anemia.

### Cell cycle defects in Cdh1-deficient erythroid cells

**Figure 4:**
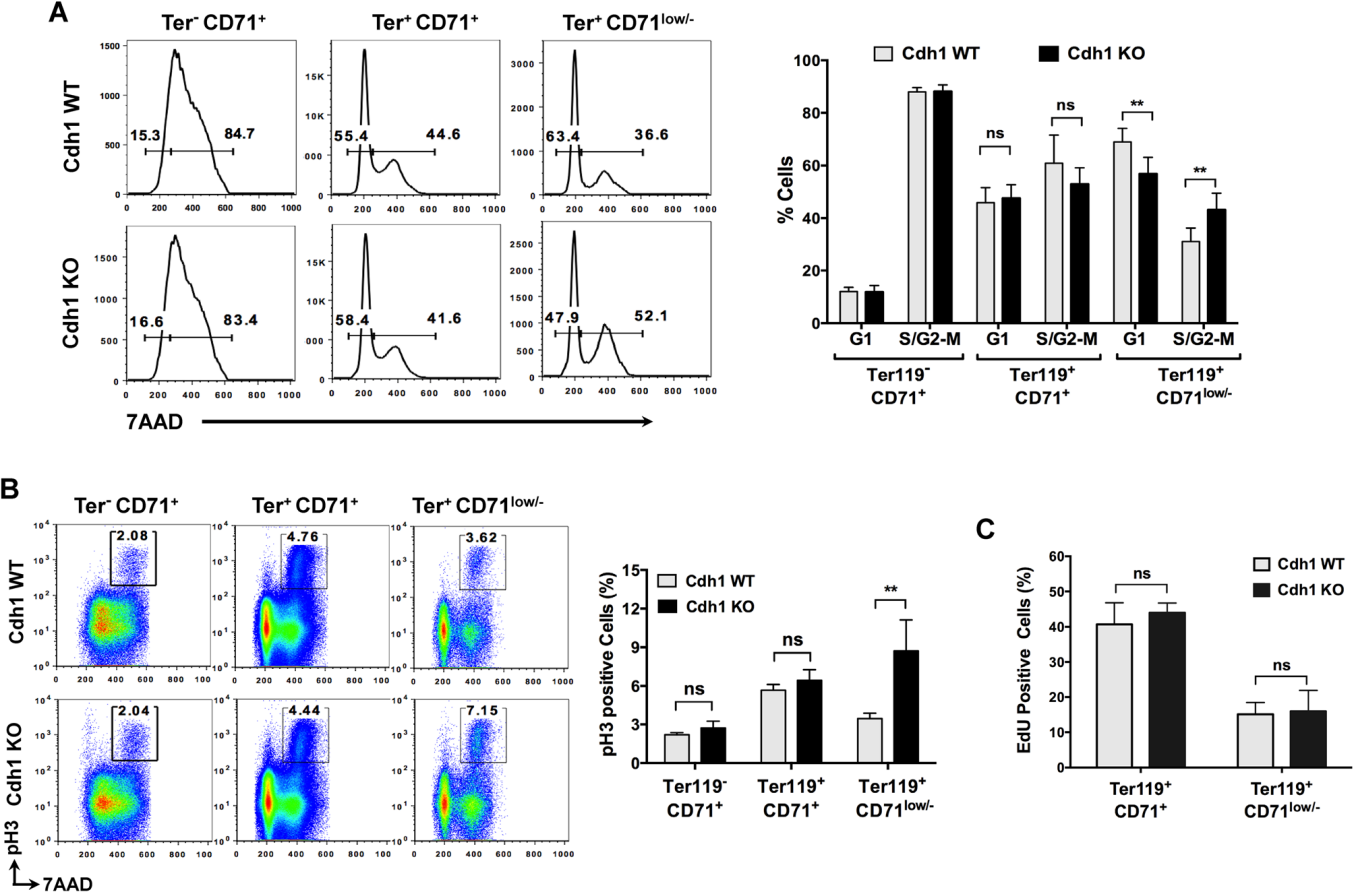
Cell cycle defects in fetal liver erythroblasts devoid of Cdh1. (**A**) Fetal liver cells from E17.5 control (Cdh1-WT) and Cdh1-null (Cdh1-KO) embryos were stained for Ter119 and CD71 surface markers and DNA content was analyzed in the indicated populations. The percentage of cells with 2N DNA content (G1) or with higher-than-2N DNA content (S/G2-M) was measured and plotted (right panel; n = 5). Representative cell cycle profiles are shown (left panels). (**B**) Quantitation of pH3 positive cells in the different erythroid populations gated based on Ter119 and CD71 expression, from control and Cdh1-deficient E17.5 fetal livers (n = 5). Examples of flow cytometry plots showing the quantified region are included on the left. (**C**) The percentage of EdU positive cells was assessed in sorted Ter119^+^ CD71^+^and Ter119^+^ CD71^low/-^ erythroblast populations from control (Cdh1-WT) and Cdh1-null (Cdh1-KO) E17.5 fetal livers (n = 4). **P < 0.01, ns (not significant) *P* > 0.05.

**Figure 5:**
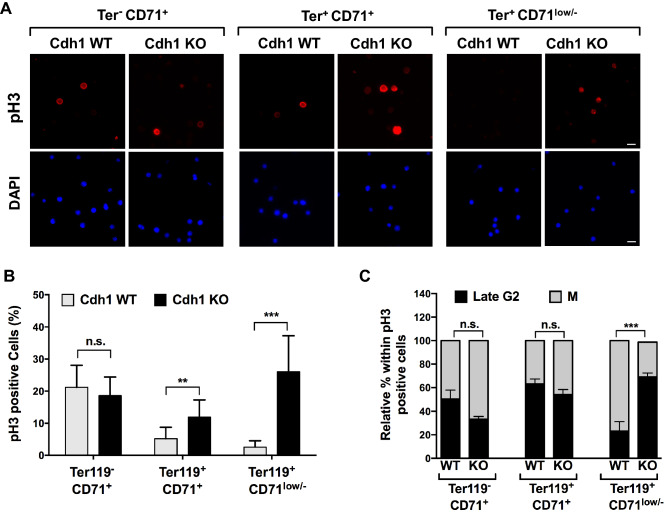
Increased presence of pH3 positive cells in maturing erythroblasts devoid of Cdh1. (**A**) The indicated proerythroblasts and erythroblast populations from E17.5 fetal livers were sorted, and expression of phospho-Histone H3 (pH3) was analyzed by immunofluorescence microscopy. Representative pictures are shown. Scale bar: 10 µm. (**B**) pH3 positive cells were quantified for both genotypes and the results of two independent experiments are summarized in the column graph. At least 950 cells of each genotype were scored in Ter119^-^ CD71^+^ and Ter119^+^ CD71^+^ populations, and at least 600 of each genotype were scored in Ter119^+^ CD71^low/-^ populations. (**C**) Within the pH3 positive cells in the indicated populations, the relative ratio of spotted (late G2) or bright and diffuse (mitosis, M) staining pattern was determined for both genotypes and plotted as stacked bars. Error bars represent s.e.m. **P < 0.01, ***P < 0.001, ns (not significant) *P* > 0.05.

As a first approach to uncover the mechanism leading to inefficient erythroid differentiation in the absence of Cdh1, we turned back to the fetal liver of Cdh1 WT and Cdh1 KO embryos and examined the cell cycle profile of the three main populations defined by Ter119 and CD71 expression. No differences were detected between WT and KO embryos in the earlier stage Ter119^-^ CD71^+^ proerythroblast population or in immature erythroblasts (Ter119^+^ CD71^+^) (Fig. 4A). However, we found a significant decrease in G1 cells and a parallel increase in cells in S or G2/M phases in the most mature erythroblast population (Ter119^+^ CD71^low/-^) in Cdh1-null fetal livers from E17.5 embryos (Fig. 4A). Similar alterations were noted in samples from E15.5 embryos (Fig. S2A). Flow cytometry analysis of phosphorylated Histone H3 (pH3), that marks cells in late G2 and mitosis, revealed an even larger difference in positively stained cells between control and Cdh1-deficient late erythroblasts (Figs. 4B and S2B). The increase in non-G1 cells and in pH3 positivity could reflect a faster proliferation rate in mature erythroblasts devoid of Cdh1 or a slower progression through G2 and/or M phases that would lead to aberrant accumulation of Cdh1 KO cells in these phases of the cell cycle. To distinguish between these possibilities we injected pregnant mice with the thymidine analogue EdU (ethynyl deoxyuridine) and assessed EdU incorporation in sorted fetal liver populations from wild type and Cdh1-deficient embryos. Only a modest increase in EdU-positive cells was detected in late-stage erythroblasts (Ter119^+^ CD71l^ow/-^) lacking Cdh1 (Figs. 4C and S2C), and the difference with control cells did not reach statistical significance. We can therefore discard elevated proliferation rate as a major contributor to the large increment in pH3 positivity. Accordingly, altered cell cycle progression through G2 and/or M must be posited to explain the higher ratio of cells with phosphorylated Histone H3 present in late erythroblast populations devoid of Cdh1. Intriguingly, quantitation of pH3 positive cells in sorted erythroid populations by immunofluorescence imaging (Fig. 5A,B) revealed a more dramatic divergence between Cdh1-proficient and Cdh1-deficient late stage erythroblasts, and uncovered previously undetected differences in immature erythroblasts. This observation would indicate that early erythroblasts are already affected by the loss of Cdh1, but the cell cycle defects seem to progressively aggravate during their maturation process. Moreover, a significant increase in the ratio of late G2 (spotted) versus mitotic (bright and diffuse) pH3 staining pattern was observed in Cdh1 KO late erythroblasts when compared with same stage Cdh1 WT erythroblast populations (Fig. 5C). Therefore, Cdh1 deficiency seems to result in delayed or impaired G2/M transition in terminally differentiating erythroblasts.

### Maturing erythroblasts accumulate DNA damage in the absence of Cdh1

**Figure 6:**
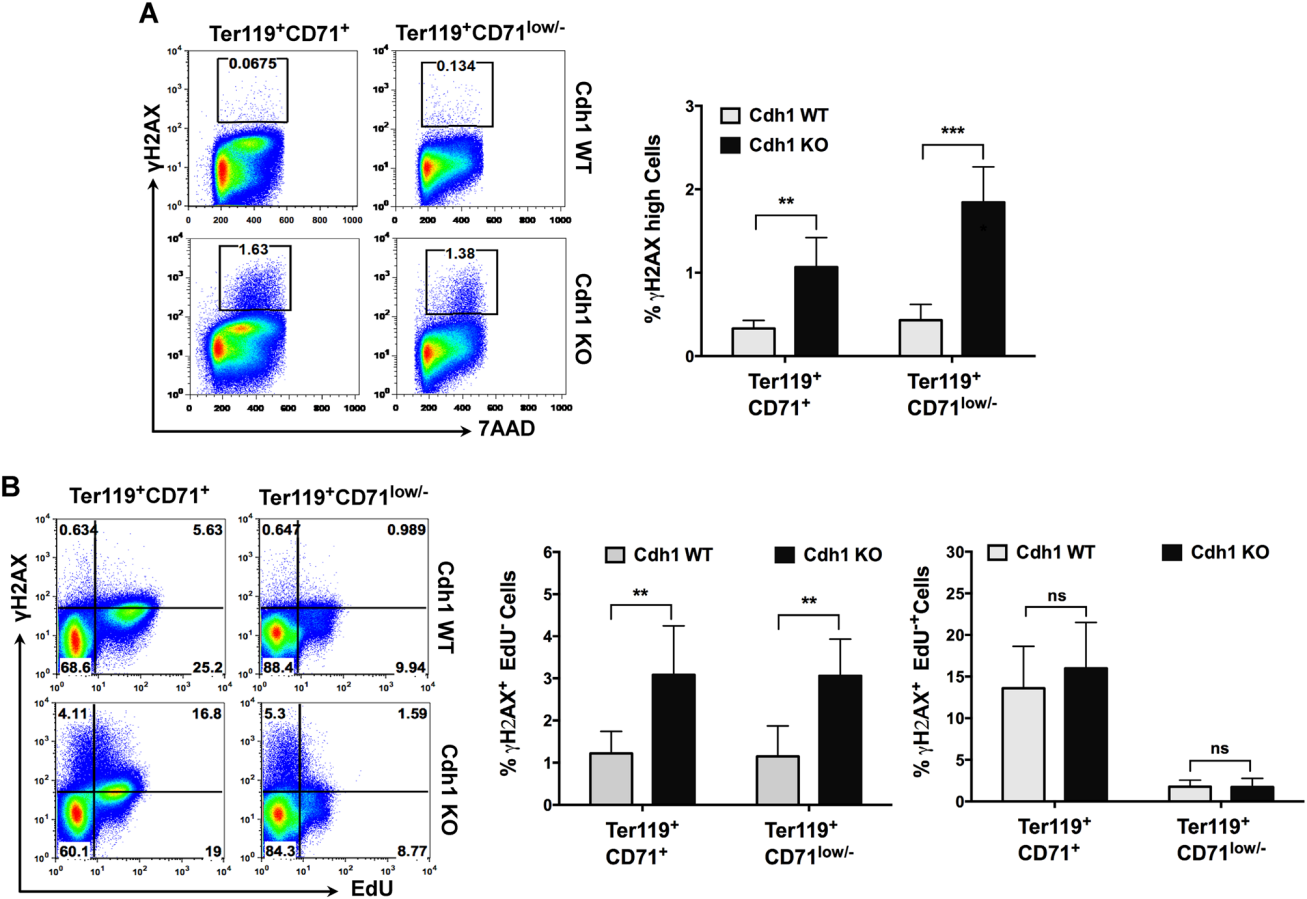
Accumulation of DNA damage in Cdh1-deficient maturing erythroblasts. Fetal liver cells from control (Cdh1-WT) and Cdh1-null (Cdh1-KO) E17.5 embryos were sorted based on Ter119 and CD71 expression and then stained for EdU, ɣH2AX and DNA content (n = 4 for each genotype). (**A**) Quantitation of cells with strong ɣH2AX staining in the indicated sorted erythroblast populations. Left panels show representative flow cytometry plots and include the gate used for quantitation (black box). (**B**) Within each of the sorted erythroblast populations, the percentage of cells positive for ɣH2AX and EdU (right column graph) or positive for ɣH2AX but negative for EdU (left column graph) was measured. The representative flow cytometry plots (left panels) show the corresponding gating. **P < 0.01, ***P < 0.001, ns (not significant) *P* > 0.05.

Delayed progression through G2 and M phases could be caused by accumulation of unrepaired DNA lesions. In this regard, Cdh1 deletion has been shown to promote genomic instability in different cell systems^12,13,16,17^. Therefore, we set out to explore whether maturing erythroblasts devoid of Cdh1 accumulate DNA damage. To that end we monitored phosphorylated H2AX (γH2AX), a common marker of DNA injury, in sorted fetal liver erythroblasts. As shown in Fig. 6A, a significantly higher percentage of cells with high γH2AX signal was present in both early (Ter119^+^ CD71^+^) and late (Ter119^+^ CD71^low/−^) Cdh1-null erythroblasts, although the most mature populations seemed to comprise more damaged cells. Interestingly, following in vivo labelling with EdU, that incorporates into replicating cells, the largest discrepancy with control samples was found in the ratio of γH2AX^+^EdU^-^ cells (Fig. 6B). Therefore, while DNA lesions are generated during S phase in both wild type and mutant cells, the differential accumulation of DNA damage due to Cdh1 loss preferentially occurs in cells that no longer incorporate traceable levels of the thymidine analogue, either because they already completed the bulk of genome duplication or because they suffer from highly inefficient replication. Importantly, most erythroblasts with intense γH2AX labelling seem to display a 4N DNA content (see cytometry plots in Fig. 6A) suggesting that more DNA breaks persist in late S and/or throughout the G2 phase. Overall, these results indicate that, in the absence of Cdh1, erythroblasts accumulate DNA lesions, particularly at their later stages of maturation. The presence of strong γH2AX immunohistochemistry staining in fetal liver sections from Cdh1-deficient embryos (Fig. S3) further confirms these observations.

### Signs of replicative stress in fetal liver cells devoid of Cdh1

**Figure 7:**
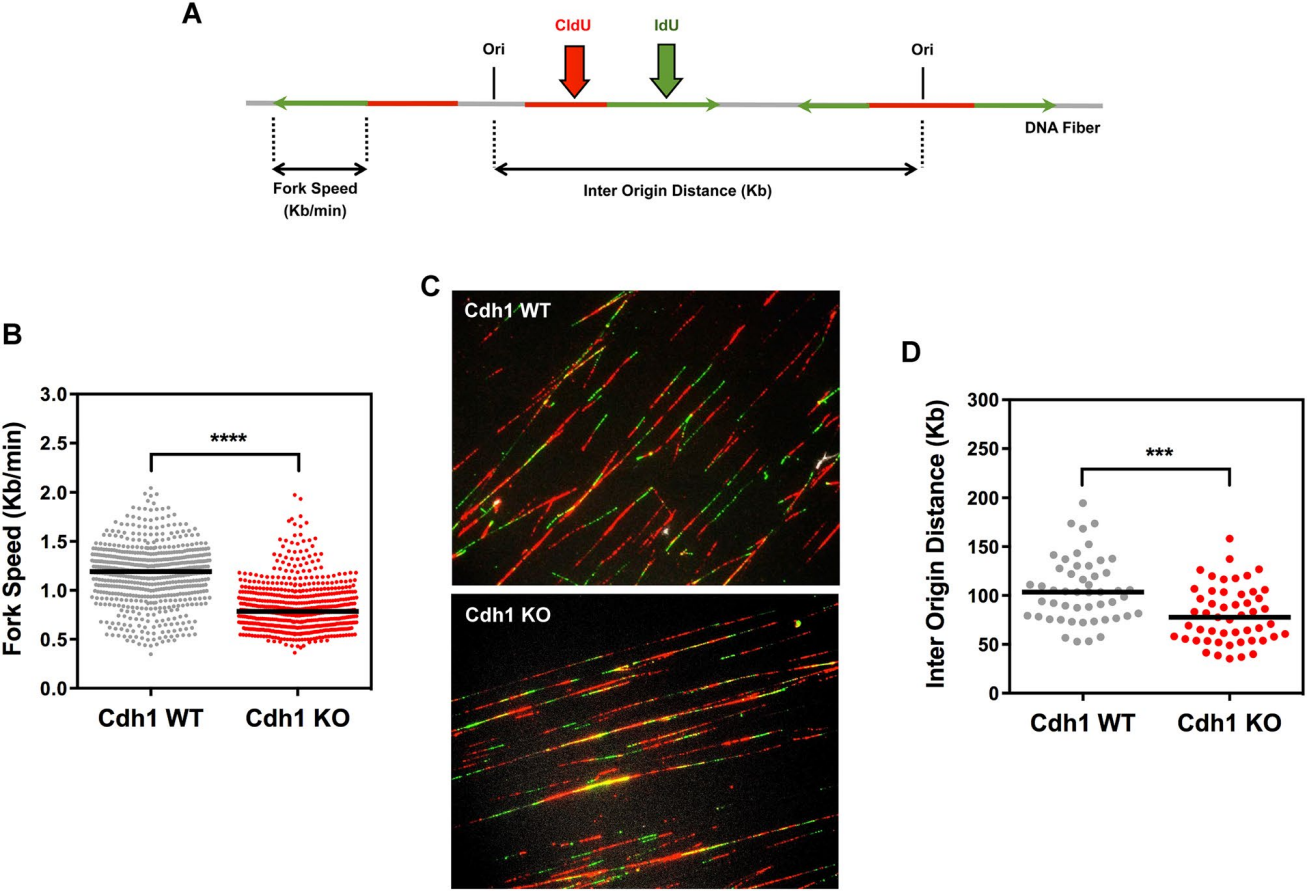
Aberrant replication dynamics in fetal liver cells in the absence of Cdh1. (**A**) Schematic drawing of a DNA fiber after pulse labeling with CldU (red) and IdU (green). Green track measurements allow quantification of fork speed. Origins are identified based on the pattern of green and red staining and the distance between two adjacent origins on the same DNA fiber defines IOD. (**B**) Replication fork rates were measured as detailed in (**A**) on stretched DNA fibers obtained from E14.5 fetal liver cells of the indicated genotype (n = 607 for WT and 601 for KO). (**C**) Representative immunofluorescence images of stretched DNA fibers from the indicated fetal liver cells showing CldU (red) and IdU (green) staining. (**D**) Distribution of IOD values from the same samples as in (**B**) (n = 50 for WT and 51 for KO). In scatter dot plots in (**B**) and (**D**) the black line indicates median value for the plotted data. ***P < 0.001, ****P < 0.0001.

Our next step was aimed at identifying the source of the endogenous DNA damage noted in Cdh1-null erythroblasts. We have previously reported that primary MEFs deficient for Cdh1 suffer from replicative stress and, as a result, accumulate DNA breaks^17^. Therefore, we hypothesized that aberrant replication was behind the observed DNA lesions in erythroid cells devoid of Cdh1. To test this idea, fetal liver cells from Cdh1 WT and Cdh1 KO embryos were labelled ex vivo with the thymidine analogues CldU (chloro-deoxyuridine) and IdU (iodo-deoxyuridine) and used to directly assess replicative parameters on stretched DNA fibers (Fig. 7A). Replication fork progression was found to be markedly slower in Cdh1-deficient fetal liver cells (median fork rate: 0.785 kb/min) than in control samples (median fork rate: 1.19 kb/min) (Fig. 7B), as illustrated by the shorter IdU tracks (Fig. 7C, green tracks). Moreover, the distance between adjacent replication origins (Inter Origin Distance, IOD) was also altered in the absence of Cdh1 and was significantly shorter in Cdh1 KO fetal liver cells (median IOD: 103.4 kb for WT and 77.59 kb for KO) (Fig. 7D). Shortened IOD is indicative of increased origin firing and is frequently associated with slow fork progression that prompts compensatory activation of dormant origins. We conclude that fetal liver cells lacking Cdh1 expression do show altered replication dynamics compatible with replicative stress. We propose such defects to be the underlying cause of the observed DNA damage accumulation and the resulting cell cycle delay at G2/M, both of which, would, in turn, restrain terminal erythroid differentiation and limit production of mature erythrocytes.

## Discussion

The E3-ubiquitin ligase complex APC/C-Cdh1 drives proteosomal degradation of a large variety of proteins involved in cell cycle control but also in other cellular functions such as differentiation, apoptosis, metabolism and DNA damage repair, among others. Important insight into the biological role of this complex has been gathered through genetic deletion of its substrate-adaptor subunit, Cdh1. In this study, we used mouse models of Cdh1 deletion to explore if this ubiquitin ligase plays a physiologically relevant role in erythropoiesis.

Initially, we detected accumulation of immature erythroblast populations and shortage of the more mature ones and of enucleated reticulocytes in fetal liver from Cdh1-deficient embryos. This phenotype was later recapitulated in embryos with a hematopoietic-restricted Cdh1 deletion, thus validating the initial observation and pointing to a lineage-specific defect. Importantly, similar alterations in the ratio of mature versus immature erythroid populations were detected in the bone marrow and spleen of hematopoietic Cdh1-deficient animals, indicating that the presence of Cdh1 is required for efficient terminal erythroid differentiation not only during embryonic development, but also postnatally. This impaired transition through the final stages of erythroid maturation results in inefficient production of red blood cells and leads to mild but persistent anemia from birth to adulthood. Moreover, we provide evidence that stress erythropoiesis in response to hemolytic anemia, is also affected by the absence of Cdh1. Other critical cell cycle regulators, such as D-cyclins^31,32^, Rb^33,34^, E2F2^28^, E2F4^35^, E2F8^36,37^, and the MCM complex^38^ have been previously shown to play relevant roles in erythropoiesis and to prevent anemia in mouse embryos and/or adult mice, underscoring the need of a tight coordination between cell cycle progression and erythroid differentiation. Interestingly, the erythroid phenotype of Cdh1 deletion we describe here is reminiscent of what was formerly reported for Rb, as they both seem to be dispensable for expansion of early erythroid progenitors but particularly important during terminal erythroid differentiation. Therefore, our findings further highlight the relevance of negative regulators of the G1/S transition (such as APC/C-Cdh1 and Rb) during the final steps leading to mature erythrocytes. As deletion of either Cdh1 or Rb on their own leads to moderate anemia compatible with life, it is tempting to speculate that both tumor suppressors work redundantly to sustain red blood cell homeostasis, and that a combined inactivation of Rb and Cdh1 would dramatically blunt erythropoiesis.

In a previous study, a conditional gene trap was employed to deplete Cdh1 from HSCs in young mice^39^, but the authors failed to detect any variation in white or red blood cell counts following Cdh1 deletion and, consequently, did not examine erythropoiesis. The divergent outcomes of both studies could rely on experimental differences such as the use of an inducible Cre line (Mx1-Cre) to acutely deplete Cdh1 in 8–12 week old mice instead of the Vav1-Cre transgene used in our approach that drives expression of the recombinase from mid-gestation onwards^40^. It is possible that the timing of Cdh1 deletion in HSCs might determine whether or not the anemia phenotype is manifested. Alternatively, Cdh1 deletion could be less efficient in their mouse model and the remaining Cdh1 expression could be sufficient to sustain effective production of red blood cells.

How does the absence of Cdh1 affect erythroblasts during their maturation to produce enucleated reticulocytes? Our analyses revealed progressively worsening cell cycle defects in maturing erythroblasts characterized by reduced presence of G1/G0 cells with no significant rise in replicating cells, and a striking increase in pH3 positivity corresponding mostly to late G2 cells. These alterations are suggestive of impaired entry into the mitotic phase that could delay or obstruct final erythroblast maturation, as effective enucleation requires prior cell cycle exit and, as has been previously shown, a G2/M arrest is not suitable for inducing nuclear extrusion^41^. Our results further suggest that the difficult G2/M transition could stem from accumulation of DNA damage in erythroblasts throughout their maturation process, as high ɣH2AX staining was detected in these cells in the absence of Cdh1. The damage seems to be particularly prominent in slow-replicating cells or cells that have already duplicated the bulk of their genome and show a 4N DNA content. We found a similar pattern of ɣH2AX staining in Cdh1-null primary MEFs that suffer from replicative stress and DNA breakage^17^. Importantly, we were able to obtain direct evidence of notably slow replication fork progression in erythroid cells devoid of Cdh1, thus pointing to stressed replication as the source of DNA lesions in maturing erythroblasts. However, we failed to detect the expected increase in the percentage of EdU positive cells in either early or late sorted erythroblast populations, although a tendency to higher ratios was observed in the absence of Cdh1. This could reflect an overall longer division cycle in Cdh1-ablated cells that might partially mask the extended duration of the S phase in those cells. Interestingly, defective neurogenesis in the absence of Cdh1 was also linked to increased DNA damage in developing mouse brain. Moreover, Cdh1-ablated mouse neural progenitors accumulated in G2 and failed to efficiently differentiate into mature neurons^21^. The DNA damage was then attributed to replication stress but the rate of replication fork movement could not be assessed in these in vivo studies. In the current work, we go one step further and show altered replication dynamics in differentiating cells lacking Cdh1 expression. In this regard, our results further support the notion that APC/C-Cdh1 is critically required during differentiation to prevent impaired replication and DNA breakage, and to ensure effective production of fully differentiated cells.

Aberrant replication due to low expression of the replicative DNA helicase MCM^38^ or to deletion of the DNA repair enzyme PARP-2 (Poly [ADP-ribose] polymerase 2)^29^ has also been shown to trigger defective erythropoiesis and cause anemia in mice. Likewise, concomitant inactivation of murine Rb and E2F8 triggers replicative stress and a partial block in terminal erythroid differentiation due to unrestrained E2F2 transcriptional activity^37^. E2F targets could also show altered expression patterns in the absence of Cdh1, as atypical repressors E2F7 and E2F8 are APC/C-Cdh1 substrates^42,43^. Moreover, both of them together with E2F1, E2F2 and E2F3A, are targeted for degradation in G2 by the E3-ubiquitin ligase SCF-Cyclin F^44–46^, and cyclin F itself is degraded following APC/C-Cdh1-mediated ubiquitination^47^. Therefore, while the final impact of Cdh1 loss on specific E2F targets is difficult to predict, the reported role of different E2F family members on erythropoiesis and their direct and indirect links to APC/C-Cdh1, suggest that an imbalanced E2F transcription could at least be partly responsible for the erythropoietic phenotype observed upon Cdh1 deletion.

The origin of inefficient replication in Cdh1-deficient erythroblasts was not addressed in the current study, but our prior work in primary MEFs determined shortage of dNTPs to be the underlying cause of replication defects in the absence of Cdh1^17^. Imbalanced levels of cellular nucleotides were previously shown to lead to ineffective erythropoiesis in Erk5-deficient mice^48^. Importantly, a number of nucleotide biosynthesis enzymes are E2F targets^49^. Hence, although alternative mechanisms to impair replication cannot be ruled out, nucleotide insufficiency caused by altered E2F transcription or by other dysregulations, could contribute to induce the observed replicative defects in maturing erythroblasts devoid of Cdh1.

Taken together our data support a novel role for the APC/C-Cdh1 ubiquitin ligase complex in facilitating terminal erythroid differentiation by limiting replicative stress and thus preventing accumulation of DNA damage and delayed G2/M transition in maturing erythroblasts. These alterations could impair progression towards fully functional erythrocytes and lead to anemia. In this regard our observations raise the intriguing possibility that reduced APC/C-Cdh1 activity might be behind some of the human diseases associated to ineffective erythropoiesis.

## Materials and methods

### Mice and treatments

To obtain Cdh1-null embryos we used previously described Cdh1 conditional knockout^13^ and Sox2-Cre transgenic^50^ mouse strain. Crosses were established between Cdh1(lox/lox) female mice and Cdh1(−/+); Sox2-Cre male mice, and the resulting Cdh1(+/lox) and Cdh1(−/Δ); Sox2-Cre embryos were referred as wild type (Cdh1 WT) and Cdh1-deficient (Cdh1 KO) respectively. To obtain mice with a hematopoietic-restricted Cdh1 deletion, we crossed Cdh1(lox/lox) males with females carrying the Vav1-iCre transgene that has been shown to drive expression of the Cre recombinase in Hematopoietic Stem Cells (HSCs) (*B6*.*Cg-Tg(Vav1-cre)A2Kio/J* The Jackson Laboratory, stock no. 008610). For experiments involving adult mice, animals of both sexes and ranging between 6 and 12 weeks old were used. To induce acute anemia, mice were injected intraperitoneally with Phenylhydrazine (PHZ) (Sigma-Aldrich) at a dose of 100 mg/kg.

Mice were kept in a C57BL6 genetic background and were housed under standard conditions in the pathogen-free animal facility of the University of Salamanca following the animal care standards of the institution. Animal procedures were approved by the Bioethics Committee of the University of Salamanca and performed according to Spanish legislation (RD1201/2005) and in compliance with the ARRIVE guidelines.

### Blood cell counts

Peripheral blood (~ 50 µl) was collected from the mandibular vein of adult mice or through exsanguination of newborn pups and erythrocyte parameters were measured on an automated instrument (Hemavet Counter HV950FS).

### Flow cytometry and cell sorting

Fetal liver cell suspensions were obtained by disaggregating in RPMI 2% FBS livers extracted from E13.5–E17.5 embryos. Erythroid populations were stained in 2% Bovine Serum Albumin (BSA) in phosphate-buffered saline solution (PBS) using Ter119-FITC (1/200; 557915), CD71-PE (1/200; 553267) conjugated antibodies from BD Pharmingen. 7-aminoactinomycin D (7-AAD, Invitrogen, 1 µg/ml) was used to exclude dead cells from analysis. To measure enucleation, cells stained for cell surface markers were incubated with 1 µM DRAQ5 (Cell Signaling Technology) for 15 min at RT. Bone marrow and spleen cells from adult mice were similarly stained for Ter119 and CD71 and CD45-APC antibodies (1/200; BD Pharmingen 559864) were included to allow negative selection of myeloid and lymphoid cells. For staging of erythroid populations based on CD44 and FSC, CD44-APC antibodies (1/200; BD Pharmingen 559250) were used together with Ter119-FITC. For cell cycle analysis a fraction of the fetal liver samples described above were fixed and permeabilized with BD Cytofix/Cytoperm™ (BD Pharmingen 554723) following the manufacturer´s instructions. Next we stained for intracellular phospho-histone H3 (Ser10) (pH3; 1/100; Millipore 06–570) followed by secondary anti-rabbit IgG A647 (1/500; Invitrogen A21244). Samples were finally resuspended in 1%BSA in PBS with RNAse (20 µl of a 100 µg/ml stock of RNase per sample) and 7-AAD (25 µg/ml) for DNA content and kept in the dark for 30 min before flow cytometry.

For quantitation of replicating cells, pregnant female mice were intraperitoneally injected with 5-ethynyl-2′-deoxyuridine (EdU, Invitrogen; 25 mg/kg) and embryos were harvested 60 min later. Fetal liver cells were stained for Ter119 and CD71 as indicated above except with Ter119-Pacific Blue antibodies (1/200; Biolegend 116232), and sorted erythroblasts populations were processed for EdU detection with a ClicK-iT EdU Flow Cytometry Assay kit (Invitrogen C10425) according to manufacturer’s instructions. The same cells were then stained with a γH2AX-A647 conjugated antibody (1/10; BD Pharmingen 560,447) and with 7AAD (25 µg/ml) for DNA content analysis.

Flow cytometry was performed on FACSCalibur or FACSAria II flow cytometers (BD Biosiences) and analyzed with FlowJo software (Tree Star). For sorting of erythroid populations a FACSAria II cell sorter (BD Biosiences) was employed.

### Immunofluorescence microscopy

Cells in suspension were plated onto coverslips previously incubated with fibronectin (2 μg/ml, Roche 10838039001) for 1 h at 37 °C in PBS. Afterwards cells were fixed and permeabilized with BD Cytofix/Cytoperm™ (BD Pharmingen 554723) for 10 min, washed twice with PBS, blocked in 2% BSA in PBS for 30 min, and then incubated in a wet chamber with primary antibodies to pH3 (1/100; Millipore 06–570) followed by secondary anti-rabbit IgG A647 (1/500; Invitrogen A21244). Finally, cells were stained with 1 μg/ml of 4′,6-diamidino-2-phenylindole (DAPI; Invitrogen D1306) for 3 min to visualize the nuclei and images were captured in a Laser Confocal Microscopy Leica SP5.

### Single-molecule analysis of DNA replication

Single cell suspensions of fetal livers were pulse-labeled with 50 μM CldU (20 min) followed by 250 μM IdU (20 min), harvested, and resuspended in 0.2 M Tris pH 7.4, 50 mM EDTA and 0.5% SDS. Stretched DNA fibers were prepared essentially as described^51^. For immunodetection of labeled tracks, fibers were incubated with primary antibodies (BD Biosciences 347580 1/100 and AbCam ab6326 1/00) for 1 h at RT and with the corresponding secondary antibodies for 30 min at RT, in a humidity chamber. DNA was stained with anti-single-stranded DNA antibody (Millipore MAB3034 1/200) to assess fiber integrity. Images were obtained in a Nikon Eclipse 90i microscope with a Plan Apo-Chromat VC 60 × objective. The conversion factor used was: 1 μm = 2.59 Kb. At least 250 tracks/sample were measured for fork rate estimation, and at least 25 fibers with two or more origins/ sample were scored for Inter Origin Distance (IOD) estimation.

### RNA isolation and quantitation

Total RNA was isolated using RNeasy Mini Kit (50) (Qiagen #74104) following the supplier’s instructions and analyzed by qRT-PCR using the Power SYBR Green RNA-to-CT™ 1-Step kit (Cat. #4389986, Applied Biosystems) and the StepOnePlus Real-Time PCR System (Cat. #4376600, Applied BioSystems) according to the supplier’s instructions. Raw qRT-PCR data were processed with the StepOne software v2.1 (Applied Biosystems) using *Gapdh* as intersample normalization control. Primers used for transcript quantitation are 5′-GTTTCAGAGATGCGGAGAACC-3′ (forward for mouse *Cdh1*), 5′-CAGGCCGTCTTTGCCATTG-3′ (reverse for mouse *Cdh1*); 5′-TGC ACC ACC AAC TGC TTA GC-3′ (forward for *Gapdh*) and 5′-TCT TCT GGG TGG CAG TGA TG-3′ (reverse for *Gapdh*).

### Immunoblotting

Bone marrow and spleen cell suspensions were lysed in Laemmli buffer and 50 µg of total protein were separated by SDS-PAGE and transferred to nitrocellulose membranes (Bio-Rad). The membranes were cut in two pieces just above the 37 kDa marker and the upper part was probed with antibodies against Cdh1 (AR38, a generous gift from Dr J Gannon, Cancer Research UK) while the lower part was incubated with anti- GAPDH antibodies (AbCam ab8245). Secondary antibodies were HRP-conjugated (Jackson Immunoresearch) and blots were developed with ECL reagents (Western Lightning Plus, Perkin Elmer).

### Statistical analysis

Unless otherwise stated all bar graphs represent mean ± s.d. of the corresponding data. Statistical significance was established based on *P* values obtained from Student´s unpaired *t* test (GraphPad Prism v6.0). The data for replication parameters (Fork Speed and IOD) were analyzed using the Mann–Whitney test.

## Supplementary Information

Supplementary Figures.

## Data Availability

The datasets used and analyzed during the current study are available from the corresponding author on reasonable request.
