# Supplementary material for: Efficient terminal erythroid differentiation requires the APC/C cofactor Cdh1 to limit replicative stress in erythroblasts
Source: Sci Rep. 2022 Jun 21;12:10489. doi: 10.1038/s41598-022-14331-6 (PMC9213546; doi:10.1038/s41598-022-14331-6)

SUPPLEMENTARY FIGURES AND FIGURE LEGENDS

Efficient terminal erythroid differentiation requires the APC/C cofactor Cdh1 to limit replicative stress in erythroblasts

Myriam Cuadrado[1,2], Javier Garzón[1,4], Sergio Moreno[1] & Irene García-Higuera[1,3]

[1] Instituto de Biología Funcional y Genómica (IBFG), CSIC / Universidad de Salamanca, 37007 Salamanca , Spain

[2] Instituto de Biología Molecular y Celular del Cáncer, CSIC/Universidad de Salamanca, 37007 Salamanca, Spain.

[3] Departamento de Biología Molecular, Instituto de Biología Molecular (IUBM) and Centro de Biología Molecular "Severo Ochoa", Universidad Autónoma de Madrid/CSIC, 28049 Madrid, Spain

[4] Present Address : Adrestia Therapeutics, Babraham, Cambridge CB22 3AT, United Kingdom

Correspondence: Irene García-Higuera , Centro de Biología Molecular Severo Ochoa, C/ Nicolás Cabrera 1, Madrid 28049, Spain (irene.garcia@cbm.csic.es) and Sergio Moreno, Instituto de Biología Funcional y Genómica, C/ Zacarías González 2, Salamanca 37007, Spain (smo@usal.es)

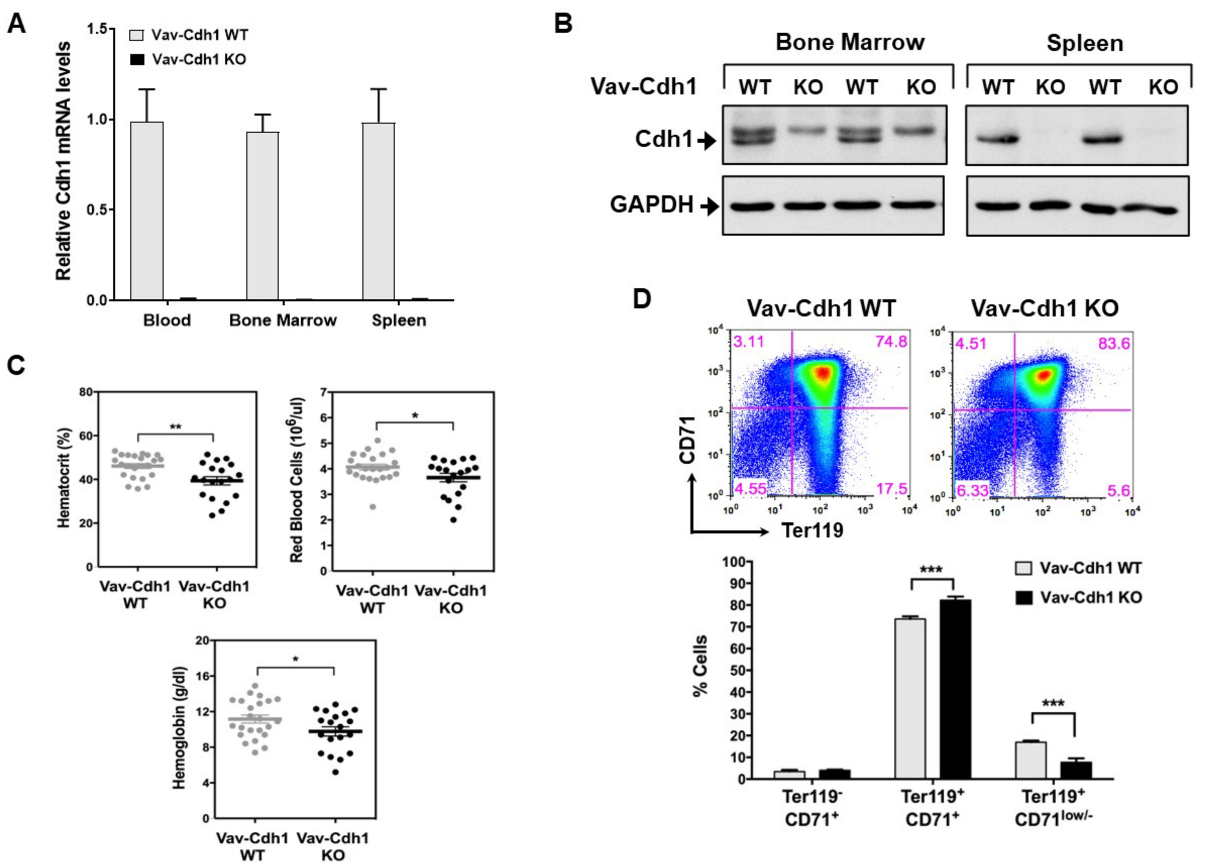

FIGURE S1: Specific deletion of Cdh1 in hematopoietic stem cells also elicits anemia at birth and defective fetal erythropoiesis. A) Relative Cdh1 mRNA levels in blood cells and hematopoietic tissues from control mice (Vav-Cdh1 WT) and mice carrying the Vav1-Cre transgene (Vav-Cdh1 KO). Each qRT-PCR reaction was performed in triplicate and the data normalized against GAPDH. An arbitrary value of 1 was given to the results obtained with control samples (n = 2 age-matched mice for each genotype). **B)** Immunoblot analysis of Cdh1 levels in the indicated tissues from the same Vav-Cdh1 WT and Vav-Cdh1 KO mice used in A. Note that in bone marrow samples an additional band with a slightly lower mobility is detected in Cdh1 immunoblots (upper left panel). The intensity of that band is not affected by the presence of the Vav1-Cre transgene and can therefore be considered a non-specific cross-reacting band. The specific, Cdh1 band is the lower band in the doublet. GAPDH is included as loading control. **C)** Peripheral blood of newborn pups with the indicated genotype was collected and red cell parameters were analyzed. The scatter dot plots show mean ± s.e.m of the corresponding data. (n = 23 for Vav-Cdh1 WT and 19 for Vav-Cdh1 KO). **D)** Erythroblast maturation in E16.5 fetal livers from control (Vav-Cdh1 WT; n = 6) and mutant (Vav-Cdh1 KO;

n = 4) embryos. Cell surface expression of Ter119 and CD71 was assessed by flow cytometry (representative plots shown in top panels) and the percentage of proerythroblasts ($Ter119^{-}CD71^{+}$), immature erythroblasts ($Ter119^{+}CD71^{+}$), and mature erythroblasts and reticulocytes ($Ter119^{+}CD71^{low/-}$) was quantified in three independent experiments and plotted (bottom). * *P* < 0,05 , ** *P* < 0,01 , *** *P* < 0,001

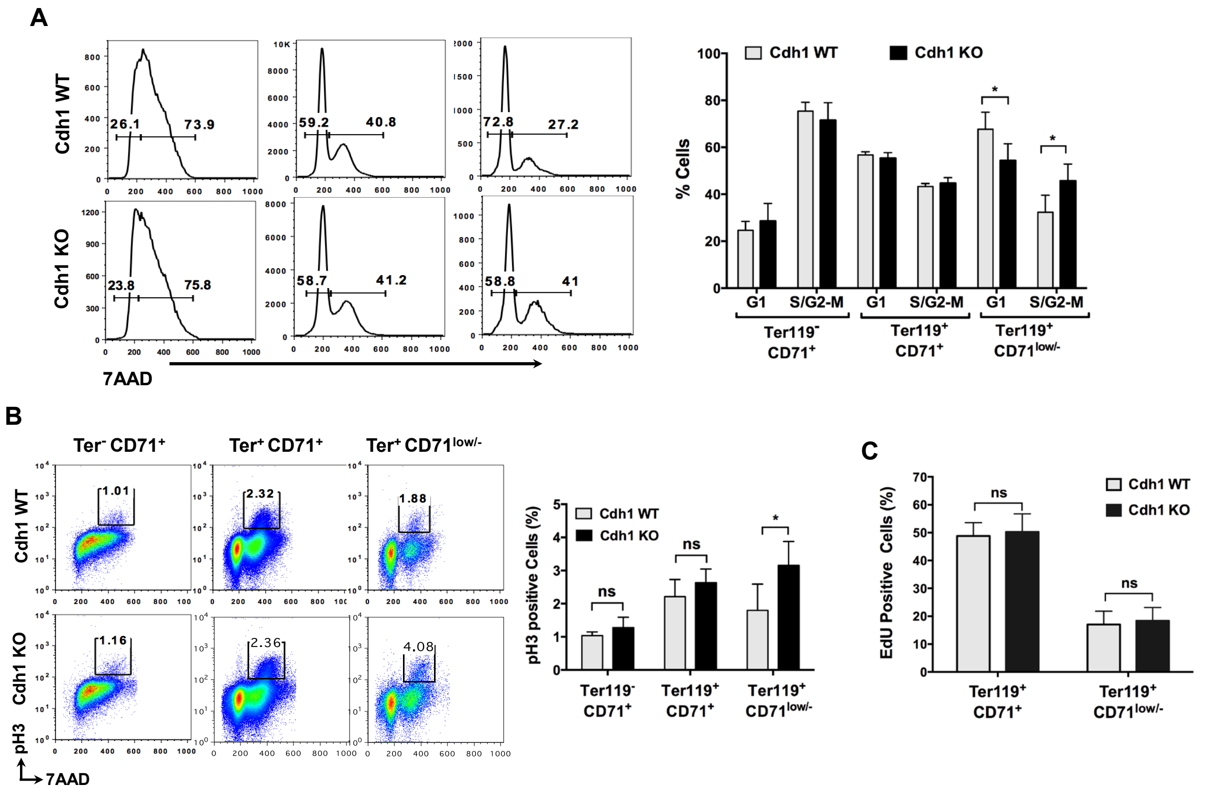

FIGURE S2: E15.5 fetal liver erythroblasts lacking Cdh1 expression accumulate in G2/M: A) DNA content was analyzed in the indicated erythroid populations gated based on Ter119 and CD71 expression and the percentage of cells in G1 phase (2N DNA content) or in S/G2-M (higher than 2 DNA content) was determined (n = 5). Representative cell cycle profiles are shown in left panels. **B)** The same populations as in A) were stained for phospho-Histone H3 (pH3) and the percentage of positively labelled cells was quantified in Cdh1 WT and Cdh1 KO samples (n = 4). Flow cytometry dot plots are included on the left with the black box showing the selected pH3 positive cells. **C)** The percentage of EdU positive cells was assessed in sorted TER119$^+$CD71$^+$ and Ter119$^+$CD71$^{low/-}$ erythroblast populations from Cdh1-null E15,5 fetal livers (Cdh1-WT) (n = 5). * P < 0,05 , ns (not significant) P > 0,05

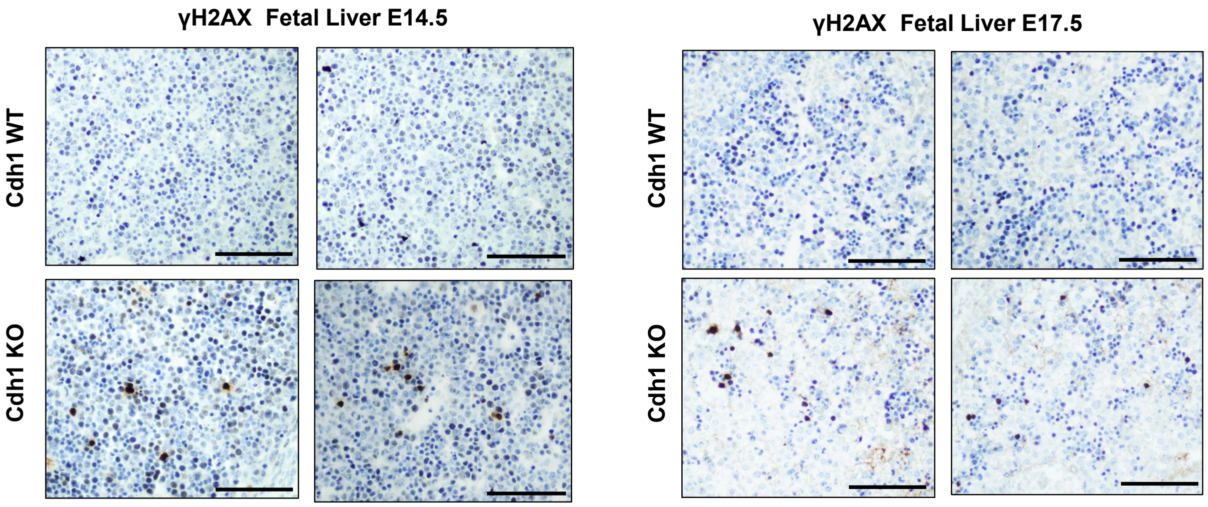

FIGURE S3: Increased ɣH2AX immunohistochemistry staining in the liver of Cdh1-deficient embryos. Liver sections from embryos of the indicated stage and genotype were stained for ɣH2AX. Representative pictures are shown. Scale bar: 100 µm

Uncropped scans of Western blots in Figure S1:

Cdh1 Blots Figure S1B - Short Exposure

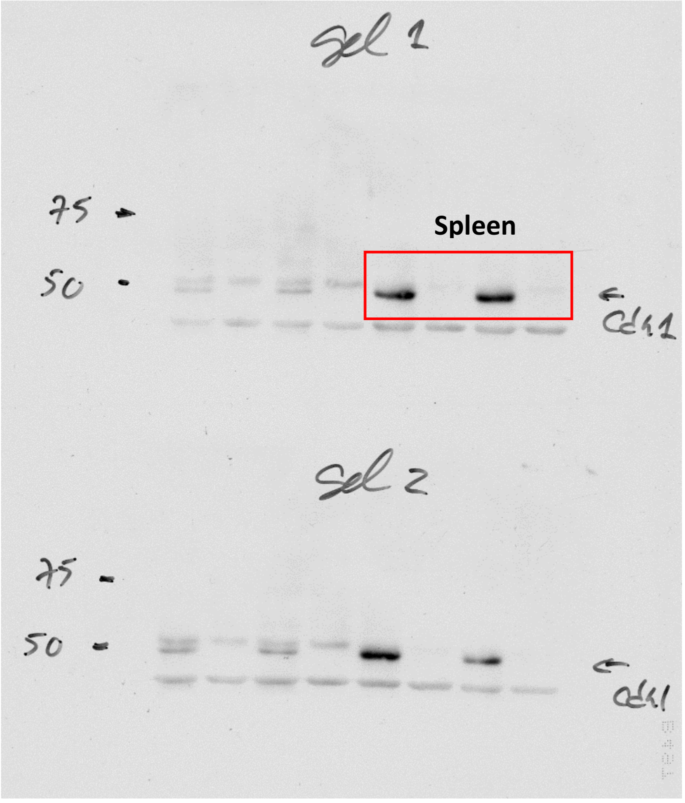

Cdh1 Blots Figure S1B – Long Exposure

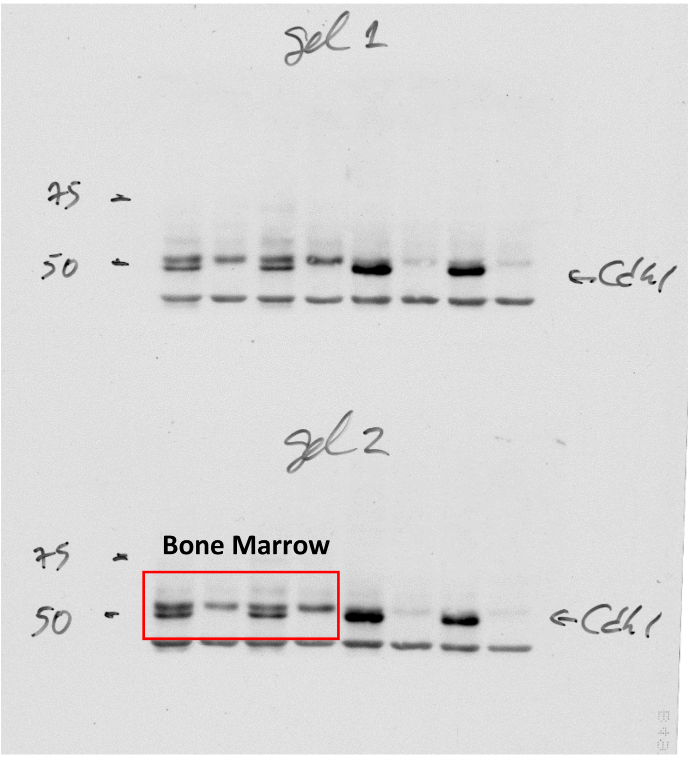

GAPDH Blots- Figure S1B

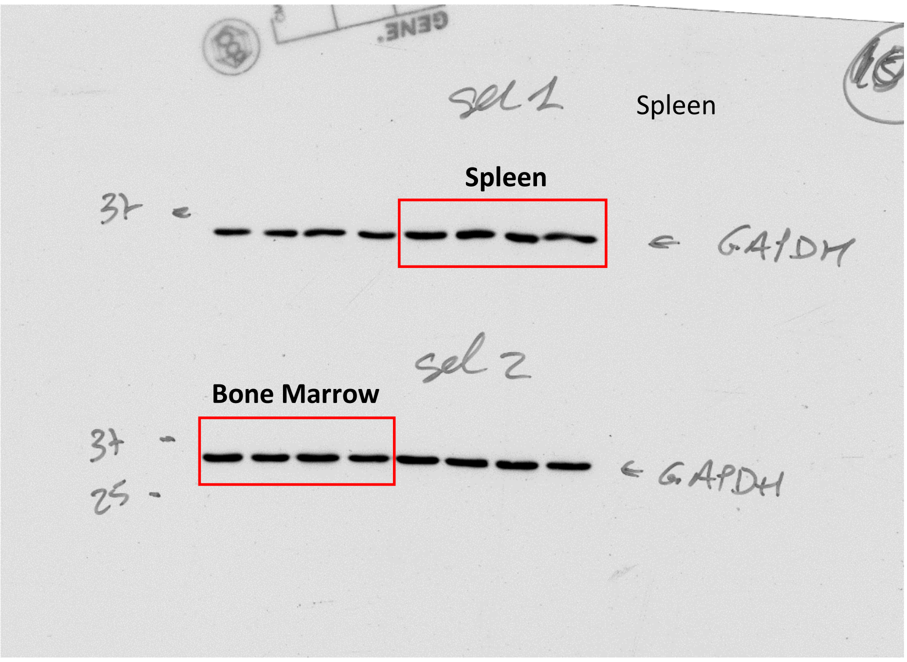

Supplement: Supplementary file 1 — Supplementary Figures. [file 41598_2022_14331_MOESM1_ESM.pdf]
